# Cytosolic Hsp60 Is Involved in the NF-κB-Dependent Survival of Cancer Cells via IKK Regulation

**DOI:** 10.1371/journal.pone.0009422

**Published:** 2010-03-23

**Authors:** Jung Nyeo Chun, Boae Choi, Kyung Wha Lee, Doo Jae Lee, Dong Hoon Kang, Joo Young Lee, In Sung Song, Hye In Kim, Sang-Hee Lee, Hyeon Soo Kim, Na Kyung Lee, Soo Young Lee, Kong-Joo Lee, Jaesang Kim, Sang Won Kang

**Affiliations:** 1 Division of Life and Pharmaceutical Science and Center for Cell Signaling and Drug Discovery Research, Ewha Womans University, Seoul, Korea; 2 Department of Life Science and College of Natural Science, Ewha Womans University, Seoul, Korea; 3 College of Pharmacy, Ewha Womans University, Seoul, Korea; 4 Division of Electron Microscopic Research, Korea Basic Science Institute, Daejeon, Korea; 5 Department of Anatomy, College of Medicine, Korea University, Seoul, Korea; Universidade Federal do Rio de Janeiro (UFRJ), Brazil

## Abstract

Cytoplasmic presence of Hsp60, which is principally a nuclear gene-encoded mitochondrial chaperonin, has frequently been stated, but its role in intracellular signaling is largely unknown. In this study, we demonstrate that the cytosolic Hsp60 promotes the TNF-α-mediated activation of the IKK/NF-κB survival pathway via direct interaction with IKKα/β in the cytoplasm. Selective loss or blockade of cytosolic Hsp60 by specific antisense oligonucleotide or neutralizing antibody diminished the IKK/NF-κB activation and the expression of NF-κB target genes, such as Bfl-1/A1 and MnSOD, which thus augmented intracellular ROS production and ASK1-dependent cell death, in response to TNF-α. Conversely, the ectopic expression of cytosol-targeted Hsp60 enhanced IKK/NF-κB activation. Mechanistically, the cytosolic Hsp60 enhanced IKK activation via upregulating the activation-dependent serine phosphorylation in a chaperone-independent manner. Furthermore, transgenic mouse study showed that the cytosolic Hsp60 suppressed hepatic cell death induced by diethylnitrosamine *in vivo*. The cytosolic Hsp60 is likely to be a regulatory component of IKK complex and it implicates the first mitochondrial factor that regulates cell survival via NF-κB pathway.

## Introduction

Mammalian cells express a number of survival genes that play the roles in inhibiting caspase activation, removing harmful oxygen radicals, defending mitochondrial function, and checking cell cycle. Among the transcription factors responsible for the induction of the survival genes, nuclear factor-κB (NF-κB) is a key element that orchestrates the complex cell survival response [1]. In particular, the NF-κB-dependent survival genes include anti-apoptotic genes, such as c-IAPs and c-FLIP, and mitochondrial safeguard genes, such as manganese-superoxide dismutase (MnSOD) and Bcl-2 family members [2], [3], [4]. A central kinase in NF-κB activation pathway is the inhibitor of κB kinase (IκB kinase or IKK) that phosphorylates the IκB protein in two amino-terminal serine residues, leading to its ubiquitinylation and proteosomal degradation and to the consequent liberation of NF-κB proteins [5]. The extracellular stimuli to activate NF-κB pathway converge to IKK [6]. Therefore, numerous efforts have been made to delineate the regulation of IKK activation. First, the phosphorylation-dependent regulation of IKK activation has been characterized [7]. The phosphorylation of two serine residues (Ser 177/Ser181 in human IKKβ) in activation T-loop is essential for activation, while the autophosphorylation of carboxyl-terminal serine cluster turns off the activation. Many kinases have been implicated to be involved in the activation phosphorylation: NF-κB-inducing kinase (NIK) [8], [9], mitogen-activated protein kinase/ERK kinase kinases 1 (MEKK1) [10], MEKK2/3 [11], [12], Hematopoietic progenitor kinase-1 (HPK1) [13], Mixed-lineage kinase 3 (MLK3) [14], TGF-β activated kinase 1 (TAK1) [15]. However, except TAK1, it is unclear how the upstream kinase activates the IKK complex. Second, the ubiquitin-dependent regulation of IKK activation has been studied for many years [15], [16]. Recently, the regulatory subunit IKKγ (or NEMO) of IKK complex has been shown to be ubiquitinated, as well as to recognize Lys63-linked polyubiquination chain on receptor-interaction protein 1 (RIP1) [17], [18]. Thirdly, the regulation of IKK activation via the interacting proteins has been characterized. The best examples are heat shock proteins. For example, Cdc37 and Hsp90 have been reported to act as additional components of the IKK complex that stabilize the complex [19], [20]. Hsp27 has been shown to interact with IKKβ in a TNF-α-dependent manner [21]. Hsp70 also interacts with IKKγ but interferes with the IKK activation [22]. Besides, the association between protein phosphatase 2Cβ (PP2Cβ) and the IKK complex has been demonstrated [23], and ELKS has also been identified as a new regulatory subunit of IKK complex that mediates the recruitment of IκB to the complex [24]. However, there is no indication of a mitochondrial protein involved in the IKK/NF-κB activation.

We have recently identified heat shock protein 60 (Hsp60) as an IKK-interacting protein. Hsp60 is the mitochondrial chaperonin that promotes the folding of the imported protein to native conformation. Nonetheless, the existence and function of Hsp60 in extramitochondrial compartments has frequently been stated [25]. For example, Hsp60 can be a ligand for cell-surface receptors like toll-like receptor [26]. Intracellularly, Hsp60 has been shown to interact with pro-caspase-3 or Bax [27], [28], [29]. However, the function of Hsp60 related to cell survival signaling has not been reported. In this study, Hsp60 was found to mediate the NF-κB-dependent survival signaling in the cells. Specifically, Hsp60 directly interacted with IKKα/β in cytoplasm and then promoted the phosphorylation-dependent activation of the kinase in response to TNF-α. Such signaling function of Hsp60 was independent of its chaperone activity, which sets Hsp60 apart from other heat shock proteins that function via stabilizing or destabilizing signaling complex [30]. We also showed *in vivo* evidence that cytosolic expression of Hsp60 protects hepatic cells against chemical-induced damages via enhancing IKK activation. Thus, this finding represents the novel pro-survival function of cytosolic Hsp60 and shed a light on understanding the function of Hsp60 in extra-mitochondrial compartments [25].

## Results

### Hsp60 interacts with IKK complex in cytoplasm

**Figure 1:**
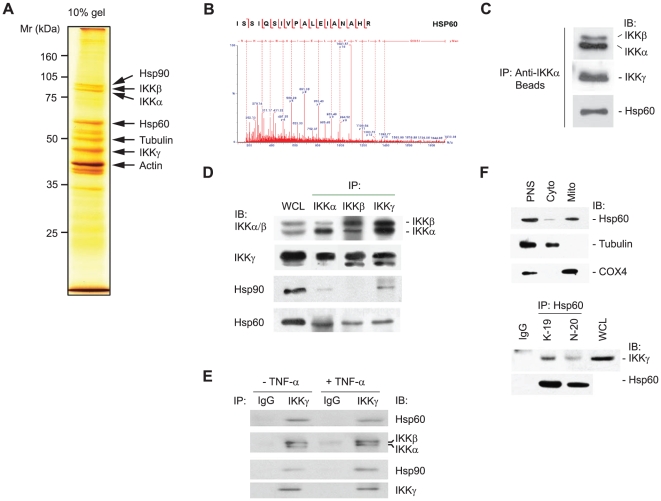
Identification of Hsp60 in IKK complex. **A**. A silver-stained polyacrylamide gel resolving the affinity-purified IKK complex. **B**. MS/MS spectra of [M+2H]^2+^ ions of the peptides derived from the protein band corresponding to Hsp60. **C**. Immunoblot (IB) analyses of IKK subunits and Hsp60 in the affinity-purified complex. **D**. Interaction of Hsp60 with IKK complex. IKK complex was immunoprecipitated (IP) from HeLa cell lysates (500 µg total proteins for each) with IKKα, IKKβ, and IKKγ-specific antibodies. The IKKα/β/γ subunits, Hsp60, and Hsp90 were immunoblotted. WCL, whole cell lysate. **E**. TNF-α-independent interaction of Hsp60 and IKK complex. **F**. Co-immunoprecipitation of Hsp60 and IKK complex in cytosolic fraction. *Upper panel*, post-nuclear supernatant (PNS), cytosol (Cyto), and mitochondria (Mito) fractions from HeLa cells were immunoblotted. COX4 and tubulin were used as mitochondrial and cytosolic markers, respectively. *Lower panel*, Hsp60 was immunoprecipitated from the cytosolic fraction using either control goat IgG or anti-Hsp60 antibodies (K-19 and N-20). Representative blots are shown (*n* = 3).

To identify an additional component, we examined the molecular composition of the latent IKK complex using a proteomic technique combining immuno-affinity purification and mass spectrometry. Briefly, the IKK complex was precipitated from the lysates of unstimulated HeLa S3 cells using anti-IKKα antibody beads, and the co-precipitated proteins were sequenced by liquid chromatography-tandem mass spectrometry. The identification of the IKK subunits and Hsp90 indicated that the immunopurification of IKK complex fairly worked (Fig. 1A). As a consequence, this proteomic study identified a heat shock protein, Hsp60, in the precipitates (Fig. 1A and 1B). The presence of the IKK subunits and Hsp60 in the precipitates was confirmed by immunoblotting (Fig. 1C). Then, we decided to investigate the biological meaning of the IKK-Hsp60 interaction.

**Figure 2:**
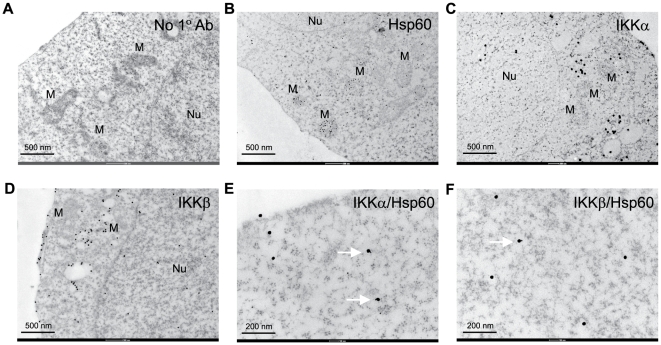
Visualization of Hsp60 and IKK interaction at a single-cell level. HeLa cells were immunoreacted with no primary (**A**), anti-Hsp60 (**B**), anti-IKKα (**C**), anti-IKKβ (**D**), anti-Hsp60/IKKα (**E**), and anti-Hsp60/IKKβ (**F**) antibodies, and then labeled with the corresponding secondary antibodies conjugated with 20 nm or 40 nm gold particles, as described in Experimental Procedure. The labeling was assessed by immuno-gold electron microscopy. Nuclei (Nu) and mitochondria (M) are indicated. *Arrows* indicate direct adherence of Hsp60- and IKK-labeled gold particles. No immunoreactive signal was seen in the sample without primary antibodies (**A**). The experiments were repeated twice with the same results, and representative results are shown.

The first step was to verify the endogenous interaction of Hsp60 and IKKs by co-immunoprecipitation experiments. When the heterogeneous IKK complexes were precipitated with antibodies against IKKα, IKKβ, and IKKγ, each of the IKK subunit-specific antibodies similarly precipitated Hsp60 (Fig. 1D). In addition, Hsp90 was also co-precipitated with IKK complex [20], [21]. This interaction was found to be unaffected by TNF-α treatment (Fig. 1E), indicating that Hsp60 is a component protein of heterogeneous IKK complexes. A reverse immunoprecipitation was then carried out with the cytosolic fraction to exclude the mitochondrial contamination. The anti-Hsp60 antibodies co-precipitated IKKγ with Hsp60, whereas control goat IgG did not (Fig. 1F), confirming that cytosolic interaction of Hsp60 and IKK. In order to visualize the virtual interaction of Hsp60 with IKKs in cytoplasm, the immunogold staining combined with the electron microscopy (EM) was performed. The immune complexes of Hsp60 and IKK with their specific antibodies were detected differently using secondary antibodies labeled with 20 nm- and 40 nm-diameter gold particles, respectively. As a result, the Hsp60-labeling gold particles were distributed throughout the cellular structures: not only in the matrix and intermembrane space of mitochondria, but also in the cytoplasm and plasma membrane (Fig. 2B). In contrast, the IKKα- and IKKβ-labeling gold particles were mainly detected in the cytoplasm (Fig. 2C and 2D), while the IKKα was also detected in the nucleus, which is consistent with the previous reports [31], [32]. Although the IKK core subunits have been shown to be found in the mitochondrial fraction [33], our data showed that the IKK-labeling gold particles were often seen in the vesicular structures rather than the mitochondria (Fig. 2C and 2D). This discrepancy might be due to the previous study done with subcellular fractionation. When the Hsp60 and IKKs were co-stained, the direct binding of 20 nm and 40 nm gold particles was clearly detected in the cytoplasm (Fig. 2E and 2F). It should be noted that not all of the IKKα and IKKβ were associated with Hsp60. These results collectively indicate that the Hsp60 directly interacts with IKK complex in the cytosol.

### Hsp60 directly interact with IKKα/β, not IKKγ

**Figure 3:**
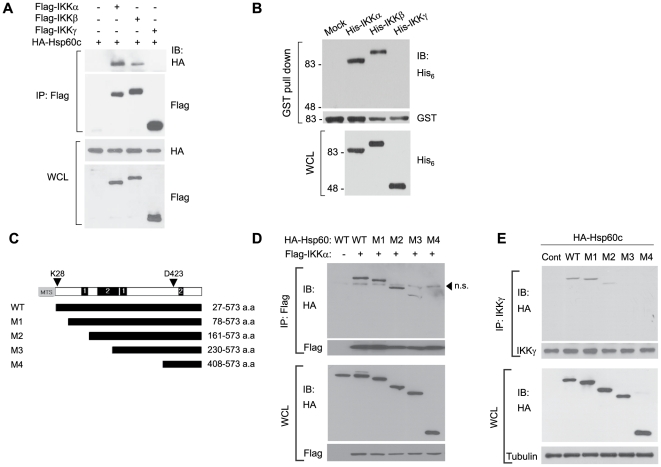
Hsp60 directly interacts with IKK complex. **A**. Direct binding of Hsp60 and IKK subunits. The 293T cells were co-transfected with Hsp60c (HA tag) and each of IKK subunit proteins (Flag tag) for 24 hrs. **B**. *In vitro* association of Hsp60 with IKKα and IKKβ. GST-fused Hsp60 proteins bound to the glutathione Sepharose beads was incubated with the lysates of Sf9 insect cells expressing His_6_-tagged IKK proteins. Hsp60 and IKKs were detected by immunoblotting for GST and HA tags, respectively. **C**. Schematic diagram showing deletion mutants of Hsp60. The putative phosphorylation sites of kinases, including PKA/PKG (1) and PKC (2), are indicated. **D** and **E**. Interaction of Hsp60 wild-type (WT) and deletion mutants with ectopically-expressed IKKα in 293T cells (**D**) or to endogenous IKK complex in HeLa cells (**E**). The control vector (C) is indicated. Representative blots and images are shown (*n* = 3). n.s., nonspecific.

Next, we analyzed the molecular interaction of Hsp60 and IKKs. To do this, a cytosol-targeted version of Hsp60 (Hsp60c), wherein the mitochondrial targeting signal sequence is deleted, was constructed. When Hsp60c was co-expressed with each of the IKK core subunits, Hsp60c interacted with IKKα and, albeit to a lesser extent, with IKKβ, but not with IKKγ (Fig. 3A). Then, an *in vitro* binding experiment using the recombinant proteins of glutathione-S-transferase (GST)-fused Hsp60 and (His)_6_-tagged IKK core subunits was assessed by a GST pull-down assay. The result, again, indicates that Hsp60 binds directly to IKKα and IKKβ, but not to IKKγ (Fig. 3B).

The molecular interaction of Hsp60 with IKK was further characterized by domain mapping experiments. Because the C-terminal deletion hampered the ectopic expression, a series of N-terminal deletion mutants of Hsp60c was tested for IKK binding via co-expression with Flag-tagged IKKα in HEK293 cells (Fig. 3C). The results showed that the N-terminal part (∼160 amino acids from N-terminus) of Hsp60 protein was shown to be dispensable for the interaction (Fig. 3D). The same result was obtained when endogenous IKK complex was immunoprecipitated from HeLa cells transfected with the Hsp60c constructs (Fig. 3E). The results fairly indicate that the core binding domain is located in the middle of Hsp60 protein.

### Hsp60 is involved in the IKK/NF-κB activation

**Figure 4:**
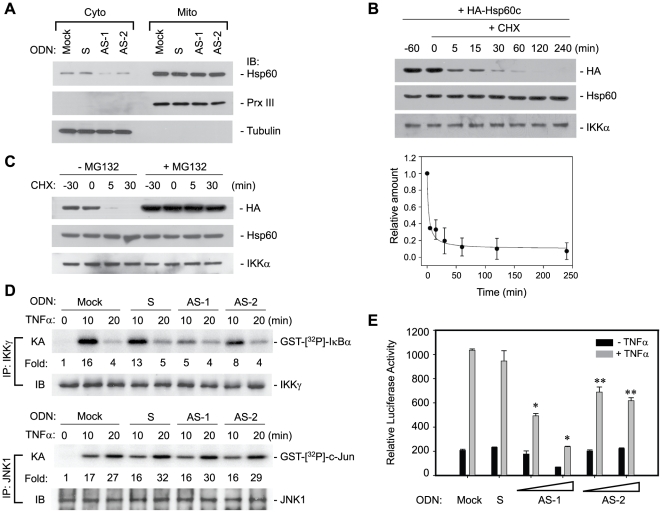
Loss of cytosolic Hsp60 diminishes IKK/NF-κB activation in response to TNF-α. **A**. Ablation of cytosolic Hsp60 by antisense ODNs. The cytosolic and mitochondrial fractions prepared from mock or ODN-transfected HeLa cells were immunoblotted. S, sense ODN; AS-1 and AS-2, antisense ODNs. The mitochondrial fraction was loaded at a volume of one-fifth of the corresponding cytosolic fraction. In particular, Prx III, which is an antioxidant enzyme present in the mitochondrial matrix, was used as mitochondrial markers to watch the nonspecific mitochondrial rupture. **B**. Half-life of ectopically-expressed Hsp60c protein (HA tag) after inhibition of protein synthesis with cycloheximide. The intensity of HA band was measured and normalized by the amount of IKKα band. Data in the graph are means ± S.D. of two independent experiments and fitted in SigmaPlot 8.0 software. **C**. Proteasome-dependent turnover of cytosolic Hsp60c protein. HeLa cells were pretreated with or without MG132 (5 µM) 30 min before cycloheximide treatment. **D**. TNF-α-induced IKK and JNK1 activation in mock or ODN-transfected cells. The *in vitro* kinase activity (KA) was averaged with the values from two independent experiments, and it is represented as a fold increase of the activity versus the unstimulated and mock-transfected cells (lane 1). **E**. NF-κB transcriptional activation in mock or ODN-transfected cells. The increasing concentration of AS-ODN (100 nM or 200 nM) was tested. The relative luciferase activity was normalized to the β-galactosidase activity and data are means ± S.D. of four independent experiments (*P<0.0001, **P<0.001 versus stimulated S-ODN-transfected cells).

Next, the biological effect of cytosolic Hsp60-IKK interaction was investigated in TNF-α-mediated NF-κB pathway. To achieve the goal, the essential step is to manipulate the level of cytosolic Hsp60 without affecting the mitochondrial one because Hsp60 deficiency is known to cause a mitochondrial functional defect [34], [35], [36]. Interestingly, a number of studies has previously reported that an antisense oligodeoxynucleotide (AS-ODN) complementary to a sequence surrounding the start codon of the human Hsp60 open reading frame actually reduces the cytosolic Hsp60 level [27], [37], [38]. We, therefore, decided to test this AS-ODN (designated as AS-1) for a selective knockdown effect. In order to exclude the possibility of non-specific action of a particular ODN sequence, we chose a second AS-ODN (AS-2) that is complementary to the region (+95∼+110 from start codon) near the 5′ end, but after mitochondrial targeting signal sequence (MTS), of Hsp60 open reading frame (Fig. S1A). The sense ODN (S-ODN) complementary to AS-1 was used as a control ODN. Since the antisense ODN is a moderate translational blocker, it did not elicit the reduction of total Hsp60 level (Fig. S1B). However, the transfection of AS-ODNs indeed selectively reduced cytosolic Hsp60 levels compared to the mock or control S-ODN without affecting the mitochondrial level (Fig. 4A). To understand this phenomenon, we hypothesized that the half-life of Hsp60 protein in two compartments may differ. To prove it, the half-life of cytosol-targeted Hsp60 (Hsp60c) was assessed after inhibition of protein synthesis. Surprisingly, the level of cytosolic Hsp60 protein was rapidly reduced (calculated *t*_½_ = 3.2 min), while total level of endogenous Hsp60 and IKKα proteins was unchanged (Fig. 4B). Moreover, this reduction was completely blocked by the treatment of a proteasome inhibitor MG132 (Fig. 4C). It is noted that the MG132 treatment also resulted in the remarkable increase of the basal level of the Hsp60c protein. Thus, this result, at least in part, explains why the level of cytosolic Hsp60 was more sensitive to AS-ODN treatment, and it additionally suggests that the level of cytosolic Hsp60 might be controlled by proteasome.

The TNF-α-induced IKK/NF-κB activation was then examined in the AS-ODN-transfected cells. An *in vitro* kinase assay showed that the transfection of AS-ODNs appreciably reduced the IKK activation in response to TNF-α by 60% compared to that of the mock or S-ODN (Fig. 4D). However, the AS-ODNs had no effect on the MAP kinase activation in response to TNF-α (Fig. 4D and Fig. S1C), revealing the specific effect of the Hsp60 AS-ODNs on IKK activation. Furthermore, the AS-ODNs almost completely abolished the NF-κB transcriptional activation in response to TNF-α, whereas S-ODN did not, compared to mock-treated cells (Fig. 4E). Owing to its knockdown efficacy, AS-1 is more potent that AS-2. However, the transfection of ODNs itself did not induce basal NF-κB activation, indicating no off-target effect of ODNs. In addition, the reductive effect of AS-ODNs on NF-κB transcriptional activity was also evident in 293T and A549 cells (Fig. S1D). An additional control experiment showed that the AS-ODNs had no affect other transcription factor activation, such as AP-1, NF-AT, and CRE (Fig. S1E).

**Figure 5:**
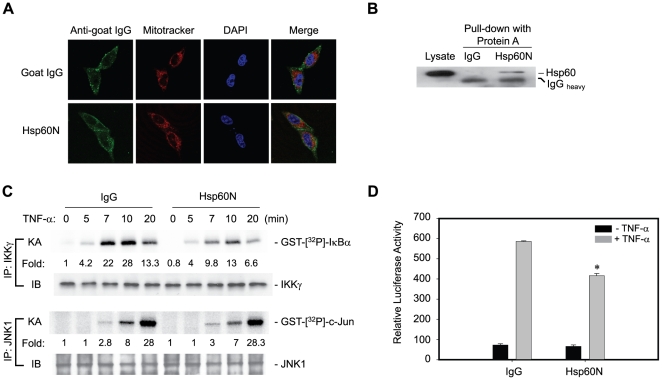
Hsp60-specific antibody blocks IKK/NF-κB activation. **A**. Transduction of Hsp60-neutralizing antibody (Hsp60N) into the cytoplasm of HeLa cells. Mitotracker Red (Molecular Probes, USA) and DAPI indicate mitochondria and nuclei, respectively. **B**. The transduced Hsp60N antibody bound to endogenous Hsp60. After antibody transfection, the HeLa cell lysates were subjected to precipitation using Protein-A Sepharose beads. The precipitated proteins were immunoblotted for Hsp60. **C**. IKK and JNK1 activation in response to TNF-α in control IgG or Hsp60N antibody-transfected HeLa cells. The *in vitro* kinase activity (KA) was averaged with the values from two independent experiments, and it is represented as a fold increase of the activity versus the unstimulated and control IgG-transfected cells (lane 1). **D**. TNF-α-induced NF-κB transcriptional activation in antibody-transfected cells (*P<0.01 versus stimulated IgG-transfected cells).

A similar study was performed by blocking the cytosolic Hsp60 using a specific antibody (Hsp60N), which has been used for immunoprecipitation and immunostaing of Hsp60 (see Fig. 1). The antibody transduction was achieved by a peptide-mediated protein delivery system [39]. The control goat IgG and Hsp60N antibody were found to be successfully delivered to cytoplasm, as being not merged with Mitotracker (Fig. 5A), and Hsp60N, but not control IgG, bound to Hsp60 (Fig. 5B). This result indicates that the delivered antibody can act as a function blocker. Then, IKK/NF-κB activation was examined in antibody-transduced cells. The Hsp60N antibody evidently reduced the IKK activation in response to TNF-α by 50% of the level obtained with the control IgG (Fig. 5C). In contrast, TNF-α-induced JNK activation was not affected, which again proves that the role of Hsp60 is specific to the IKK activation. Consistently, the Hsp60N antibody significantly reduced the transcriptional activity of NF-κB (Fig. 5D). The data collectively conclude that cytosolic Hsp60 promotes the TNF-α-induced IKK/NF-κB signaling.

### Ectopic expression of cytosol-targeted Hsp60 sufficiently promotes IKK/NF-κB activation

**Figure 6:**
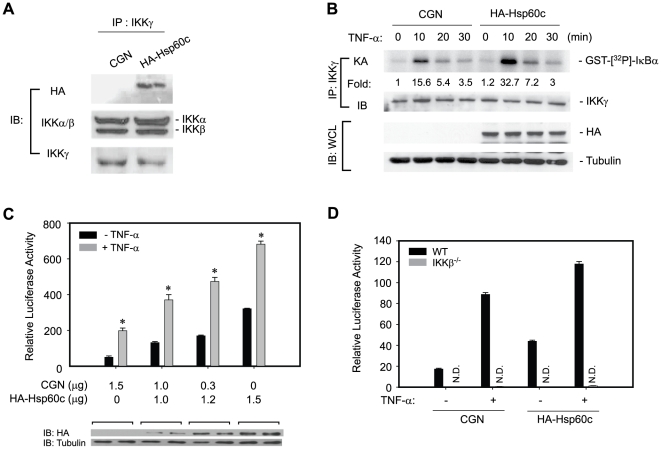
Cytosol-targeted expression of Hsp60 promotes TNF-α-induced IKK/NF-κB activation. Cells were transfected with either control (CGN) or Hsp60c-encoding plasmid (HA tag) for 24 hrs and then treated with TNF-α. **A**. Incorporation of ectopically-expressed Hsp60c (HA tag) in IKK complex. **B**. TNF-α-induced IKK activation in HeLa cells. **C** TNF-α-induced NF-κB activation in HeLa cells (*n* = 4, *P<0.0001 versus unstimulated counterpart). **D**. TNF-α-induced NF-κB activation in the transfected IKKβ^−/−^ 3T3 cells (*n* = 4, *P<0.001 versus stimulated CGN-transfected cells, N.D. not detected).

Conversely, the role of cytosolic Hsp60 in IKK/NF-κB pathway was addressed by over-expression of cytosol-targeted Hsp60c. The ectopically-expressed Hsp60c was found to associate with the IKK complex (Fig. 6A) and markedly enhanced the IKK and NF-κB activation in response to TNF-α (Fig. 6B and 6C). It should be noted that the ectopic expression of Hsp60c marginally induced the basal IKK and NF-κB activation. The effect of Hsp60c expression in NF-κB activation was completely abolished in IKKβ-deficient cells (Fig. 6D), indicating that the regulatory activity of cytosolic Hsp60 is IKK-dependent. In addition, the ectopic expression of Hsp60c did not enhance either JNK activation or the activation of other transcription factors such as AP-1, CRE, and NF-AT (Fig. S2). This result indicates that increasing the cytosolic Hsp60 level augments TNF-α-induced IKK/NF-κB activation.

### Hsp60 regulates IKK phosphorylation at the activation T-loop

**Figure 7:**
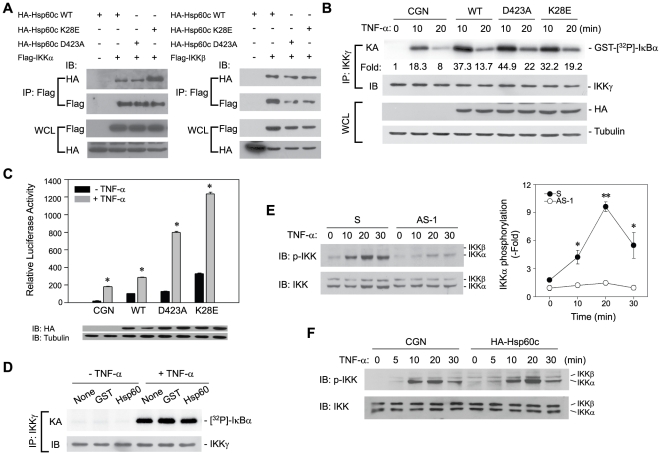
Cytosolic Hsp60 regulates IKK phosphorylation independently of chaperone activity. **A**. Association of Hsp60c wild-type (WT) and mutants with IKKα and IKKβ. The indicated proteins were co-expressed in 293T cells as shown in Fig. 3A. **B** and **C**. IKK (**B**) and NF-κB transcriptional activation (**C**) in cells expressing Hsp60c wild-type and chaperone-inactive mutants. The kinase and reporter activities were analyzed as described in Fig. 4 (for reporter assay, *n* = 6, *P<0.0001 versus unstimulated counterpart). **D**. *In vitro* kinase activity of IKK in the presence of recombinant Hsp60 protein. The IKK complex was immunoprecipitated from HeLa cell lysates and incubated with or without the indicated GST proteins (20 µg each) in the kinase reaction buffer for 10 min before the kinase reaction. **E**. Serine phosphorylation of IKKα/β in HeLa cells transfected with AS-1 ODN. Data in the graph are means ± S.D. (*n* = 3, *P<0.02, *P<0.001). **F**. Serine phosphorylation of IKKα/β in Hsp60c-expression HeLa cells. A representative blot is shown (*n* = 3).

To understand the mechanism underlying the regulatory action of Hsp60 in IKK/NF-κB activation, several experimental approaches were attempted. To determine whether the chaperone activity of Hsp60 is required, the two amino acid residues that are known to be critical for the chaperone activity of Hsp60 were considered. One is a lysine residue (K28), which is involved in the oligomerization of Hsp60 protein [40], [41]. The other is an aspartate residue (D423), which is an active site residue for ATPase activity [42], [43]. Thus, the Hsp60c mutants, wherein K28 and D423 are substituted with glutamate and alanine respectively, were constructed. The co-transfection experiment showed that both mutants interacted with IKKα and IKKβ as well as or perhaps even better than the wild type (Fig. 7A). The IKK activation in response to TNF-α in the Hsp60 mutant-expressing cells was similar to that in the wild type (Fig. 7B), indicating that such loss-of-function mutations did not affect the IKK-enhancing activity. Furthermore, TNF-α-induced NF-κB transcription was even enhanced in the Hsp60 mutant-expressing cells at approximately 4–6 times higher than that in the vector control (Fig. 7C). The enhancing effect of the mutants was clearly IKKβ-dependent, as tested again in IKKβ-deficient 3T3 cells. Thus, this experiment using the loss-of-function mutants strongly suggest that the cytosolic Hsp60 functions independently of chaperone activity in IKK/NF-κB activation.

One of the IKK-interacting protein, ELKS, has been shown to mediate the IκB recruitment to IKK complex [24]. To test this mode of action, the recombinant Hsp60 protein was directly added into the IKK kinase reaction, where the activated IKK complex is incubated with full-length human IκB as a substrate. The *in vitro* kinase activity of the activated IKK toward IκB was not affected by the presence of Hsp60 protein (Fig. 7D), indicating that Hsp60 is not involved in the interaction of IKK and its substrate IκB.

Lastly, a direct involvement of cytosolic Hsp60 in IKK activation was addressed by examining the activation-dependent serine phosphorylation in the T-loop of IKKα/β. The AS-ODN transfection markedly abolished the TNF-α-induced phosphorylation of IKK at Ser178/181, indicating that the phosphorylation-dependent IKK activation was impaired (Fig. 7E). Conversely, the ectopic expression of Hsp60c resulted in an increase of IKK phosphorylation (Fig. 7F). Overall, the data indicate that the cytosolic Hsp60 is involved in the phosphorylation-dependent IKK activation, rather than the chaperone-dependent stabilization of IKK complex.

### Cytosolic Hsp60 affects the NF-κB target gene expression and cell survival

**Figure 8:**
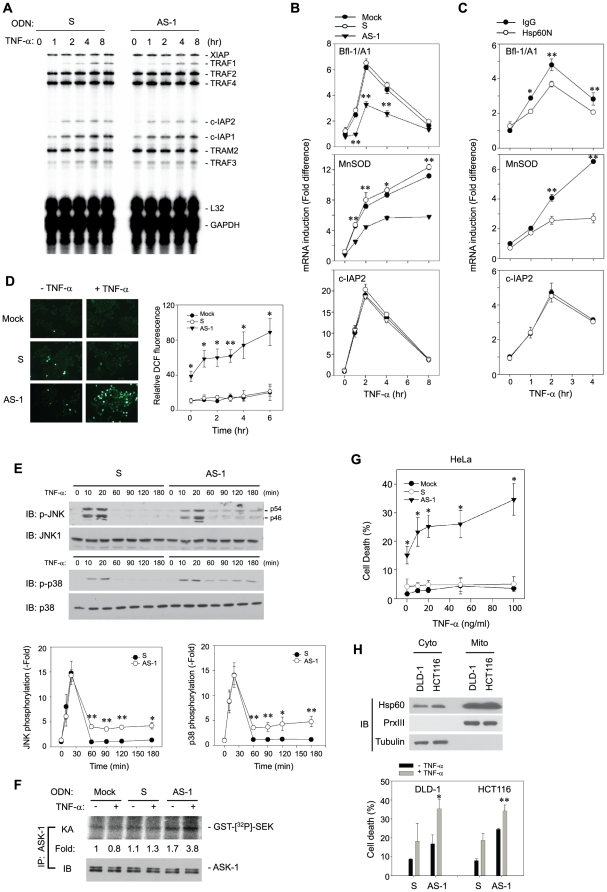
Loss of cytosolic Hsp60 induces cell death in response to TNF-α by inducing ROS and ASK-1 activation. **A**. RNase protection assay for induction of anti-apoptotic genes in ODN-transfected cells. The autoradiogram shown is a representative of three independent experiments. **B** and **C**. QPCR for induction of endogenous NF-κB target genes in ODN-transfected cells (**B**) and antibody-transfected cells (**C**) (*n* = 3, *P<0.01, **P<0.001). **D**. TNF-α-mediated production of cellular ROS in ODN-transfected cells. The representative images are shown. Data are means ± S.D. of fold increase versus untreated mock cells of the relative DCF fluorescence (*n* = 4, *P<0.05, **P<0.001). **E**. JNK and p38 MAPK activation in ODN-transfected cells. Representative blots are shown. Data in the graphs are means ± S.D. of the intensities of phospho-JNK (p46) or phospho-p38 bands that had been normalized by the corresponding non-phospho-bands (*n* = 3, *P<0.05, **P<0.001). **F**. ASK-1 activation in ODN-transfected cells. The kinase activity (KA) was averaged with the values from two independent experiments, and presented as a fold increase of the activity versus the unstimulated and mock-transfected cells (lane 1). **G**. TNF-α-induced cell death in mock or ODN-transfected HeLa. Data are means ± S.D. (*n* = 3, *P<0.01). **H**. TNF-α-induced cell death of colon carcinoma cells transfected with ODNs. The level of Hsp60 in subcellular fractions is shown (*Upper*). Data in the graph are means ± S.D. (*n* = 3, *P<0.05, **P<0.01). Cell death was analyzed by FACS after staining with annexin V-fluorescein isothiocynate and propidium iodide.

To determine the significance of cytosolic Hsp60-mediated regulation of the IKK/NF-κB pathway, we examined the expression of NF-κB target genes in ODN-transfected cells. When the expression of anti-apoptotic genes was screened by a RNase protection assay [44], the expression of TRAF1, c-IAP1, and c-IAP2 were not affected by AS-ODN transfection (Fig. 8A). This was unexpected but led us to postulate a possibility that some select target genes related to mitochondrial protection can be affected. To test this, we looked at the induction of several target genes, including antioxidant proteins (ferritin heavy chain and MnSOD) and Bcl-2 members (Bcl-2, Bcl-X_L_, Bfl-1/A1). Interestingly, the AS-ODN significantly diminished the induction of only MnSOD and Bfl-1/A1 expression in response to TNF-α (Fig. 8B). The Hsp60N antibody also significantly reduced the induction of these genes (Fig. 8C). It was again confirmed that the induction of the c-IAP2 expression was not affected in either case. Thus, the results indicate that the regulation of the IKK activation by cytosolic Hsp60 influences the expression of select NF-κB target genes.

We next wondered whether such regulation of select target genes has an impact on cell survival. Since there is a possibility that MnSOD and Bfl-1/A1 function to suppress the mitochondrial-derived reactive oxygen species (ROS) [2], [45], the level of cellular ROS was examined in ODN-transfected cells using an oxidation-sensitive fluorescence dye, CM-H_2_DCFDA. The AS-ODN transfection induced a marked increase of cellular ROS in response to TNF-α treatment in a time-dependent manner, compared to mock or S-ODN transfection (Fig. 8D). Since the enhanced ROS level is linked to cell death via the sustained JNK activation [46], the sustained activation of stress-activated protein kinases, JNK and p38 MAPK, was examined. Unexpectedly, the activation of both JNK and p38 MAPK were found to be clearly sustained in AS-ODN-transfected cells (Fig. 8E). The ASK-1 MAP3K is known to be responsible for the sustained activation of JNK and p38 in the ROS-mediated cell death [47]. Indeed, the ASK-1 activation was significantly induced in AS-ODN-transfected cells (Fig. 8F). As a consequence of this signaling pathway including ASK-1 activation, the AS-ODN resulted in a marked induction of TNF-α-induced cell death in HeLa cells, whereas the mock or S-ODN did not at all (Fig. 8G). Likewise, the AS-ODN enhanced TNF-α-induced cell death in colon carcinoma cell lines, which showed significant level of cytosolic Hsp60 (Fig. 8H). It should be noted that the AS-ODN transfection by itself resulted in the basal activation of ROS, ASK1, and cell death. Along with the evidence that the Hsp60c overexpression induced the basal IKK/NF-κB activation (see Fig. 6), cytosolic Hsp60 seems likely to direct cell survival in resting cancer cells. Collectively, our results suggest that the selective regulation of MnSOD and Bfl-1/A1 expression by cytosolic Hsp60 sufficiently influences cell survival via suppressing mitochondrial ROS burst.

### Cytosolic Hsp60 protects host cells against stressed conditions

**Figure 9:**
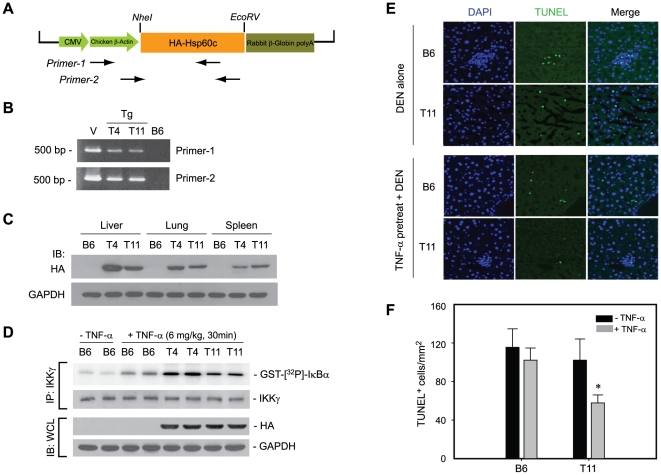
Cytosolic Hsp60 protects hepatic cells *in vivo* from stress-induced apoptosis via IKK activation. **A**. Schematic representation of transgenic vector containing HA-tagged human Hsp60c. **B**. Identification of two transgenic mouse lines (T4 and T11). Genomic PCR was performed using two different sets of PCR primers, both of which were specific to Hsp60 transgene. The transgenic vector (V) and C57BL/6j (B6) mouse genomic DNA were used as positive and negative control, respectively. **C**. Expression of Hsp60c protein in various tissues of transgenic mice. The tissue homogenates were immunoblotted using anti-HA antibody. **D**. IKK activation in the liver of control B6 mice or HA-Hsp60c-expressing transgenic mice (T4 and T11) intravenously injected with TNF-α. **E** and **F**. DEN-induced cell death in the liver of control and transgenic mice primed with or without TNF-α, as measured by TUNEL assay. The representative images (**E**) are shown. The quantified data in the graph (**F**) are means ± S.E.M. of the number of TUNEL-positive cells per unit area (*n* = 3, * *P*<0.01 versus paired stimulated one).

Next, the pro-survival activity of cytosolic Hsp60 was investigated *in vivo*. To do this, transgenic mice expressing Hsp60c were generated (Fig. 9A and 9B). The Hsp60c protein was successfully expressed in various tissues, including liver, spleen, and lung (Fig. 9C). The IKK activation was markedly enhanced in the Hsp60c-expressing transgenic mice compared to the control B6 mice when TNF-α was intravenously injected (Fig. 9D). The result indicates that the cytosolic Hsp60 enhanced the TNF-α-induced IKK activation *in vivo*. We then sought an animal model wherein the IKK/NF-κB-dependent cell survival is involved: the diethylnitrosamine (DEN)-induced hepatocyte death [48]. The apoptotic cell death of hepatocytes was indeed increased after DEN injection in four-week-old male mice (Fig. S3). Therefore, the DEN-induced hepatic cell death was examined in Hsp60c-expressing transgenic mice that had been primed with or without TNF-α. TUNEL staining of the liver tissue sections showed that the hepatic cell death was significantly reduced in Hsp60c-expressing transgenic mice compared to control mice (Fig. 9E and 9F). The data indicate that the cytosolic Hsp60 prevents the stress-induced cell death *in vivo* by promoting IKK/NF-κB activation.

## Discussion

The present study demonstrates that the cytosolic Hsp60 interacts with the IKK complex and enhances its activation. This signaling action of cytosolic Hsp60 affects the induction of the two NF-κB target genes, Bfl-1/A-1 and MnSOD, and thus cell survival. Since Bfl-1/A-1 functions as a tBid and Bak antagonist [2], [4] and MnSOD eliminates the superoxide anion inside mitochondria [49], these two genes are ultimately critical for controlling mitochondrial-derived ROS. Indeed, ablation of cytosolic Hsp60 results in the increase of cellular ROS, which in turn triggers the sustained activation of JNK/p38 via ASK-1 and finally induces the cell death, in response to TNF-α. Given that the mitochondrial ROS are causally linked to various human diseases such as cancer, vascular damage, and a variety of degenerative diseases via the accumulation of cellular damage, this new pro-survival circuitry mediated by cytosolic Hsp60 may be of particular important progression of these diseases.

The transcriptional regulation of select NF-κB target genes by cytosolic Hsp60 is attractive issue but unsolved in the present study. The RNase protection assay and the microarray experiment (∼24,000 RefSeq-based probe sequences per array, Illumina expression chip) were failed to demonstrate the broad effect of the cytosolic Hsp60 on expression profile of NF-κB target genes. Instead, the quantitative real-time PCR demonstrated the expressional change of the two select target genes, MnSOD and Bfl-1/A1, both of which are known to be related to the mitochondrial function. Although this odd phenomena were hardly investigated in the study, some experimental data can provide a clue. 1) Hsp60 was shown to bind preferentially to IKKα rather than to IKKβ in the *in vitro* protein binding assay. 2) Direct interaction of Hsp60 and IKKα, but not IKKβ, in the nucleus was often observed in the immunogold staining. IKKα is known to regulate NF-κB transcriptional activity in the nucleus [31], [32] rather than IκB phosphorylation [50]. Supposedly, when the Hsp60-IKKα complex translocate to nucleus, the Hsp60 may directly guide IKKα to the promoter of particular gene set (e.g, MnSOD and Bfl-1/A1). This is similar to the way exemplified in the regulation of select p53 target genes by human cellular apoptosis susceptibility (hCAS) protein [51]. Otherwise, it may be possible that the Hsp60 modulates the ability of IKKα or presumably IKKβ as well, to phosphorylate the other substrates such as RelA, SMRT, and histone H3 [52], which thus indirectly affects the NF-κB transcription. For eample, the IKKα-mediated phosphorylation of SMRT corepressor allows the recruitment of NF-κB to promotors [53], and IKKα-mediated phosphorylation of histone H3 affects the chromatin structure [31], [32]. However, our inciting speculation needs an investigation.

On the other hand, the functional significance of Hsp60-IKKα interaction was also investigated in non-canonical NF-κB signaling, RANK-induced osteoclastogenesis [54]. IKKα-mediated NF-κB pathway is essential for RANK-induced osteoclastogenesis [55], [56]. As shown in Fig. S5, the reduction of cytosolic Hsp60 expression using antisense ODN (AS-1) clearly suppressed the RANK-induced osteoclast formation from the bone marrow-derived macrophage/monocytic (BMM) cells, as quantified by TRAP assay. Since TNF-α-induced osteoclast formation [57] was also markedly suppressed, this result indicates that cytosolic Hsp60 can function in the NF-κB activation pathway via interacting with non-canonical as well as canonical IKK complex.

Since Hsp60 is exclusively targeted to mitochondria after translation, the cytosolic Hsp60 is presumed to be of mitochondrial origin. In fact, the mitochondrial release of Hsp60 in staurosporine-treated Jurkat T cells has been shown [29]. We also observed the same result in staurosporine-treated HeLa cells (Fig. S4). Since the Hsp60 protein was detected in the mitochondrial intermembrane space in immunogold EM data, we believe that this pool of Hsp60 protein can be released. This is similar to the case of pro-apoptotic mitochondrial factors. For example, AIF is a mitochondrial inner membrane protein (62-kDa in mature form) and is released after proteolytic shedding to a 57-kDa form upon proapoptotic stimuli [58], [59]. HtrA2/Omi (51-kDa serine protease) is released from the mitochondrial intermembrane space [60], [61]. In addition, the fact that the antisense-ODN (AS-1) used in the study targets MTS-encoding region of Hsp60 mRNA transcript but reduces the level of cytosolic Hsp60 is also supportive.

Hsp60 once located in the cytosol exhibited the faster turnover compared to the mitochondrial one. Its rapid turnover is controlled in a proteasome-dependent manner, but it is unlikely due to the structural instability because the cHsp60 protein lacking MTS sequence was successfully expressed and purified in soluble form. Nonetheless, since we cannot exclude the possibility of natural mitochondrial targeting error, the investigation on how mitochondrial Hsp60 is released and whether the turnover of cytosolic Hsp60 depends on ubiquitination is on-going.

Cytosolic Hsps such as Hsp70 and Hsp90 are known to broadly participate in cell signaling [30]. As a chaperone protein, they stabilize or destabilize the large signaling protein complexes. Since the interaction of Hsp60 and IKK complex was unaffected by TNF-α stimulation, the mode of Hsp60 action was initially thought to be the same. However, our results shown suggest a unique feature of the signaling function of cytosolic Hsp60. Briefly, the polymerization-defective or ATPase-inactive mutant of Hsp60 enhanced IKK/NF-κB activation much more effectively than the wild-type Hsp60 did (see Fig. 8). This suggests that Hsp60 likely functions as a non-chaperonic monomer rather than a chaperone polymer in augmenting NF-κB activation. Some experimental evidences also favorably support the possibility of non-chaperonic function of cytosolic Hsp60: 1) the introduction of the Hsp60N neutralizing antibody did not affect the amount of IKK core subunits in the lysate and immunoprecipitate, and 2) the AS-ODN did not affect the amount of IKK core subunits in the high-molecular-weight fractions (>600 kDa) from gel filtration chromatography (data not shown). These results indicate that the cytosolic Hsp60 is not involved in the stability of the IKK complex. Rather, our study clearly demonstrates that cytosolic Hsp60 is involved in the regulation of IKK phosphorylation in the activation T-loop. Therefore, the cytosolic Hsp60 may act on either enhancing the interaction of the IKK complex with an upstream kinase or the oligomerization-dependent auto-phosphorylation [52]. In any case, it can be a novel mechanism of the IKK activation in collaboration with the ubiquitin-dependent mechanism.

In summary, cytosolic presence of Hsp60 is frequently detected and play signaling roles in the cell types, e.g. cardiac myocytes and hepatocytes [37], [62], [63]. And also there are attempts to approach anti-cancer therapy through understanding the presence and function of Hsp60 proteins in the extra-mitochondrial compartments [25]. Our study demonstrates for the first time that, by directly interacting and regulating IKK activation, the cytosolic Hsp60 serves as survival guidance controlling mitochondrial-derived ROS through NF-κB target gene expression. Therefore, either antagonizing the interaction of Hsp60 with IKK or reducing the level of the cytosolic Hsp60 could be an additional therapeutic tool in anti-inflammatory strategy.

## Materials and Methods

### Reagents

Antibodies to IKKα (B-8), IKKγ (FL-419), Hsp90 (H-114), Hsp60 (K-19 and N-20), IκBα (C-21), JNK1 (C-17), ASK-1 (H-300 and F-9), glutathione S-transferase (B14), and goat IgG were purchased from Santa Cruz Biotechnology (Santa Crus, US); Anti-Flag antibody (M2) was purchased from Sigma; Anti-hexahistidine antibody was obtained from Qiagen; Antibodies to phosphor-IKK, IKKα, and IKKβ were from Cell Signaling Technology; Normal mouse and rabbit IgG were from Amersharm Bioscience; Anti-cytochrome c antibody were from BD Pharmingen; Antibodies to Peroxiredoxin III (Prx III), MnSOD (2AI), Hemagglutinin epitope (HA), and GAPDH were provided by AbFrontier (Seoul, Korea); Recombinant human TNF-α was purchased from Invitrogen (Grand Island, USA). The phosphorothioate oligodeoxynucleotides (ODNs), including the antisense and sense sequences, were synthesized by Hokkaido System Sciences Co. (Hokkaido, Japan). The full-length human IκB protein was a kind gift of W. Jeong (Ewha Womans University, Korea) [64].

### Plasmids

The full-length cDNA of human Hsp60 was obtained from the National Genome Information Center (Daejon, Korea). A truncated form of Hsp60, designated as Hsp60c, which lacks a mitochondrial targeting sequence (MTS; amino acids 1–26 based on human sequence), was amplified by PCR and subcloned into the pCGN-HA (a kind gift of Dr. W. Herr, Cold Spring Harbor Laboratory) and pGEX-4T1 (Amersham) vectors, by which the HA-tagged and GST-fused Hsp60c expression plasmids, respectively, were constructed.

CRE-, NF-AT-, and AP1-dependent (pAP1_7x_-Luc) firefly luciferase reporters were obtained from Stratagene. Luciferase reporter plasmids harboring the IFNβ-derived NF-κB enhancer sequences [65] was a kind gift of S. Y. Lee (Ewha Womans University, Korea). Human IKK α, β, and γ cDNAs [66], [67] were subcloned into the pCMV2-FLAG or baculovirus expression vector pFastBac-HTa (Invitrogen). The pFastBac contructs, encoding each of IKKα, β, and γ, were used for production of high-titer recombinant baculovirus stocks (∼1×10^7^ pfu/ml), according to the manufacturer's protocol. The pPuro plasmids encoding human Bcl-2 or Bcl-X_L_ were kindly provided by D.Y. Shin (Dankook University, Korea) [68]. The plasmid pGEX-4T1-SEK1 (K129R) [69] was used for production of GST-SEK1(K129R) recombinant protein. Site-directed mutagenesis was performed using a QuikChange mutagenesis kit (Stratagene).

### Immuno-affinity purification of IKK complex and ESI-q-TOF tandem mass spectrometry

HeLa S3 cells (20 ml packing volume from 20-*l* suspension culture) were gently lysed in 200 ml of lysis buffer A (20 mM HEPES (pH 7.5), 150 mM NaCl, 1 mM EDTA, 2 mM EGTA, 1% Triton X-100, 10% glycerol, 1 mM AEBSF, 1 mM Na_3_VO_4_, 5 mM NaF, 10 ug/ml aprotinin and leupeptin). The lysate (2 grams of total protein) was precleared with agarose beads alone for 1 hr, and then incubated overnight with anti-IKKα-conjugated agarose beads (2 mg/ml IgG, Santa Cruz Biotechnology). After extensively washing four times with the lysis buffer, the beads were loaded onto a column and rinsed twice with phosphate-buffered saline. The precipitated proteins were eluted twice with 1 ml of 0.1 M glycine buffer (pH 2.5). The protein eluates were immediately neutralized by adding 1 M Tris HCl buffer (pH 8.0) and separated on a 10% denaturing gel. The gel was subsequently stained with silver nitrate, and the silver-stained spots were subjected to in-gel trypsin digestion with minor modifications as follows. Briefly, the gel spots were excised with a scalpel and destained by washing with 15 mM K_4_Fe(CN)_6_, 50 mM sodium thiosulfate. The gel pieces were crushed, dehydrated by adding acetonitrile, rehydrated by adding 10–20 µl of 25 mM ammonium bicarbonate with 10 ng/µl of sequencing grade trypsin (Promega), and incubated at 37°C for 15–17 h. The peptides in the supernatant were transferred to a new tube and extracted twice by adding 50 µl of a solution containing 60% acetonitrile and 0.1% trifluoroacetic acid. The extracted solutions were pooled and evaporated to dryness in a SpeedVac vacuum centrifuge. The tandem mass spectral (MS/MS) analysis for peptide sequencing was done with nano flow reversed-phased HPLC/ESI/MS with a mass spectrometer (Q-TOF Ultima™ global, Waters Co. UK). Peptides were separated by using a C_18_ reversed-phase 75µm i. d.×150 mm analytical column (3 µm particle size, Atlantis™ dC18, Waters) with an integrated electrospray ionization SilicaTip™ (±10 µm, New Objective, USA). In detail, 5µl of peptide mixtures were dissolved in buffer A (water/ACN/formic acid; 95∶5∶0.2, v/v), injected onto a column and eluted by a linear gradient of 5–80% buffer B (water/ACN/formic acid; 5∶95∶0.2, v/v) over 120 min. Samples were desalted on a line prior to separation using a trap column (i.d. 0.35×50 mm, OPTI-PAK™ C_18_, Waters) cartridge. Initially, the flow rate was set to 200 nL/min by a split/splitless inlet and the capillary voltage (3.0 keV) was applied to the HPLC mobile phase before spraying. Chromatography was performed online using the instrument's control software MassLinx of Q-TOF Ultima™ global. The mass spectrometer was programmed to record scan cycles composed of one MS scan followed by MS/MS scans of the eight most abundant ions in each MS scan. MS parameters for efficient data-dependent acquisition were intensity (>10) and number of components (3∼4) to be switched from an MS to MS/MS analysis. Following positive identification, all identified peptides from database search (Mascot) were excluded in the next run analysis until full sequence coverage was obtained. Database analyses using the database search programs including Mascot (global search engine), Proteinlynx 2.1 (Waters Co., UK) and MODi (Korea, http://modi.uos.ac.kr/modi/), provided almost full sequence coverage on selective exclusion monitoring. MS/MS spectra were matched against amino acid sequences in SwissProt. Precusor ion mass corrections and a fragment ion mass tolerance of 0.2 Da were used to consider as 2 missed cleavages.

### Immunoelectron microscopy

The HeLa cells (1×10^7^ cells) were harvested and fixed for 1 hr, at room temperature in 0.1 M cacodylate buffer (pH 7.2) that contained 0.5% glutaraldehyde. After rinsing with cold distilled water, they were dehydrated through an ethanol treatment series at 4°C. They were infiltrated with LR White resin (London Resin, Berkshire, England) at 4°C and embedded in LR White resin in a gelatin capsules (Nisshin EM, Tokyo, Japan). Polymerization of the resin was carried out at 50°C for 24 hrs. Serial sections (120–200 sections per one sample), 70 nm in thickness, were attached to formvar-coated nickel grids. Sections were incubated in 50mM glycine for 5 min at room temperature. After rinsing with PBS, sections were incubated in 3% BSA for 30 mins at room temperature. Then, they were incubated with primary antibodies (goat anti-human Hsp60 (SC-1722), mouse anti-human IKKα (SC-7606), mouse anti-IKKβ (SC-8014), diluted 1∶100 in PBS) for 2 hrs, at room temperature. After washing five times with Tween-PBS (PBS plus 0.5% Tween-20), sections were treated with 20 nm- and 40 nm-diameter colloidal gold conjugated to anti-goat and anti-mouse IgG + IgM antibodies, respectively (BB International, UK, diluted 1∶20 in PBS) for 2 hrs, at room temperature. The sections were washed three times with Tween-PBS and then washed three times with distilled water. Sections were stained with 4% uranyl acetate for 5 mins and with lead citrate for 5 min. To examine the specificity of the primary antibody, a treatment of sections was performed with the same procedure without of the primary antibody. For double staining, antibody reactions were repeated with the second set of primary and secondary antibodies. Finally, samples were observed with a Tecnai G2 Spirit Twin transmission electron microscope (FEI Co., USA) and a JEM ARM 1300S high-voltage electron microscope (JEOL, Japan).

### Subcellular fractionation

The subcellular fractions for immunoprecipitation were acquired by differential centrifugation. Briefly, the HeLa cells (2×10^7^ cells) were harvested, rinsed twice with ice-cold PBS, and resuspended in 1 ml of a homogenization buffer (20 mM HEPES (pH 7.5), 0.5 mM EDTA, 0.5 mM EGTA, 2 mM MgCl_2_, 25 mM KCl, 1 mM AEBSF, 1 mM Na_3_VO_4_, 5 mM NaF, 5 µg/ml aprotinin, and 5 µg/ml leupeptin) containing 0.25 M sucrose. After rupturing the cells using a glass Dounce homogenizer, the post-nuclear supernatants were obtained from the homogenate by centrifugation at 750 *g* for 10 min. The supernatants were separated into pellets (mitochondrial fraction) and supernatants (cytosol fraction) by centrifugation at 15,000 *g* for 15 min. For ODN transfected cells, the subcellular fractions were obtained using the ProteoExtract subcellular proteome extraction kit (Roche). The purity of each fraction was verified by selective markers: α-tubulin as a cytosolic marker, inner membrane protein cytochrome c oxidase 4 (COX4) and matrix protein peroxiredoxin III (Prx III) [70] as mitochondrial markers. For best comparison, the mitochondrial fraction was loaded at a volume of one-fifth of the corresponding cytosolic fraction.

### *In vitro* binding assay with recombinant proteins

The Sf9 insect cells were infected with each of recombinant baculovirus stocks harboring IKKα-, IKKβ-, and IKKγ- encoding bacmids. The insect lysates expressing (His)_6_-tagged IKKs were incubated with 1.0 µg of GST-Hsp60 proteins pre-bound to glutathione-Spharose beads (Amersham Pharmacia Biotech) at 4°C for 2 hrs. The beads were washed again three times with a cold lysis buffer A. The proteins bound to the beads were eluted by boiling in a SDS sample buffer and then subjected to immunoblot analyses as indicated in Fig. 1c.

### Transfection

The ODNs (200 nM, unless indicated) was transfected for 24 hrs using Oligofectamine™ reagent (Invitrogen, USA). The plasmid transfection was achieved using Fugene-6 reagent (Roche, USA). The antibody was tranduced using a Chariot™ protein delivery kit (Active Motif Co., USA), according to the manufacturer's instruction.

### Immunoprecipitation and *in vitro* kinase assay

The HeLa cells were treated with or without TNF-α (10 ng/ml) for the indicated time periods, rinsed once with cold PBS, and lysed in the lysis buffer A. The cell lysates were precleared with 10 µl of protein A/G agarose beads (Amersham Biosciences) for 1 hr. The cleared lysates were incubated with 2 µg of Hsp60, IKKα, IKKβ, or IKKγ antibodies for 3 hrs and mixed with 20 µl of protein A/G agarose beads. The lysates were further rotated overnight at 4°C. The beads were washed three times with 1 ml of lysis buffer A. The final protein precipitates were subjected to immunoblot analyses. The immune complexes were visualized by using an enhanced chemiluminescence kit (Amersham Biosciences, USA).

For the *in vitro* kinase assay, the IKK, JNK1 or ASK-1 was immunoprecipitated with anti-IKKγ (FL-419) or anti-JNK1 (C-17) or anti-ASK-1 (H-300) antibody, respectively. The beads containing the IKK complex or JNK1 were washed twice with lysis buffer and further twice with a kinase buffer (20 mM HEPES, pH 7.4, 5 mM MgCl_2_, 10 mM β-glycerolphosphate, 1 mM Na_3_VO_4_, 2 mM NaF, and 1 mM dithiothreitol), and then incubated in 40 µl of a kinase buffer containing 10 µM ATP, 0.6 µCi [γ-^32^P] ATP, and 2 µg of either GST-IκB (1–54) or GST-c-Jun or GST-SEK1 (K129R) at 30°C for 30 min. The reaction was stopped by adding 20 µl of 3×SDS sample buffer. After boiling, the half of reaction mixture was resolved on a 10% denaturing gel and the radioactivity was detected by autoradiography. The other half of reaction mixture was used for immunoblotting of the immunoprecipitated kinase proteins (note: anti-ASK-1 antibody (F-9) for detecting ASK-1).

### Measurement of Intracellular ROS

Intracellular ROS generation was assessed with an oxidation sensitive fluoresce dye, 5,6-chloromethyl-2′, 7′-dichlorodihydrofluorescein diacetate (CM-H_2_DCFDA, Molecular Probes, USA) as described [71]. The HeLa cells (3×10^5^) were plated on 35-mm dishes and transfected with ODNs for 24 hrs. The cells were then deprived of serum for 6 hrs and stimulated with TNF-α in phenol red-free media for the indicated periods of time. After stimulation, the cells were quickly rinsed with Krebs-Ringer solution and incubated for 5 min with 5 µM CM-H_2_DCFDA. The DCF fluorescence was collected for 10 seconds with an inverted Axiovert200 fluorescence microscope (Zeiss). The relative DCF fluorescence was obtained by averaging the fluorescence intensities of the 60–80 cells in each image using ImageQuant™ software (GE Healthcare). Note that the detached round cells were omitted from quantification.

### RNase protection assay

ODN-pretreated HeLa cells were treated with or without TNF-α (10 ng/ml) for the indicated times. The total RNA was extracted with Trizol (Invitrogen). The ribonuclease (RNase) protection assay was performed according to the manufacturer's protocol (BD PharMingen). Briefly, the human apoptosis template set hAPO-5 was labeled with [α-^32^P]-uridine triphosphate. The RNA (10 µg) and 6×10^5^ cpm of the labeled probes were subjected to hybridization. After the RNase treatments, the protected probes were resolved on 5% urea-polyacrylamide gel and detected by autoradiography.

### Quantitative PCR (qPCR)

Total RNA was extracted using Trizol reagent (Invitrogen) from the HeLa cells stimulated with TNF-α for indicated periods of time. RNA (1.5 µg) was reverse transcribed using ImProm-II RT system (Promega). The real-time PCR was performed using specific primers in the presence of SYBR Green (Applied Biosystems) inside a fluorescent temperature cycler (ABI Prism 7000 sequence detection system, Applied Biosystems). The fluorescence signals were quantified by a comparative cycle threshold method. The actin mRNA was used for an endogenous control.

### Transgenic mice generation

The HA-tagged human Hsp60c lacking mitochondrial signal sequence was PCR-amplified and subcloned into pCAGGS transgenic (Tg) vector, which contains the chicken β-actin promoter, using *NheI* and *EcoRV* sites. The HA-Hsp60c Tg construct was linearized by digestion with *SalI* and *PstI* and then microinjected into eggs from C57BL/6j females. The transgenic founders were genotyped as described below. Two of the six positive transgenic lines, designated T4 and T11, were chosen for this study. The tail DNA was used for genotyping. In brief, mouse tails were incubated overnight in 100 mM Tris, pH 8.0, 0.5 mM EDTA, 200 mM NaCl, 0.2% SDS, and 100 µg of proteinase K at 55°C. DNA was extracted with phenol∶chloroform∶isoamyl alcohol (25∶24∶1) and precipitated with isopropanol. Genomic PCR was performed by using two primer sets (set 1: forward primer, 5′-ATGGCTTCTAGCTATCCTTATG-3′, and reverse primer, 5′-GTAGCAACCTGTGCAATTTCTTC-3′; set 2: forward primer, 5′-CTGCTAACCATGTTCATGCC-3′ and reverse rimer, 5′-ACAAGTTTAGCTCCAATGTTTTTGTA-3′). All the experiments were performed with 4-week-old males.

### Analysis of apoptotic cells in DEN-induced liver damage

The four-week-old male mice were injected intravenously with phosphate-buffered saline (PBS, pH 7.4) or TNF-α (6 µg/kg) via the lateral tail vein 6 hrs before intraperitoneal administration of DEN (10 mg/kg). After 48 hrs of DEN treatment, animals were sacrificed and rapidly perfused with PBS followed by 4% paraformaldehyde. The livers were removed and frozen in OCT embedding medium, and then a series of tissue sections (10 µm in thickness) was obtained in cryostat (Leica). The sections were incubated in 50 µl of terminal deoxynucleotidyl transferase-mediate uridine 5′-triphosphate-biotin nick-end labeling (TUNEL) fluorescent reaction mixture (In situ Cell Death Detection Kit, Roche Diagnostics) for 60 mins at 37°C in a dark chamber, washed and subsequently counterstained with 4′,6′-diamidino-2-phenylindole (DAPI, 1 µg/ml, Sigma) for 30 mins. The sections were mounted using the Vectashield mounting medium and examined using a LSM510 confocal laser-scanning microscope (Carl Zeiss, Germany). TUNEL-positive cells were counted and averaged from three tissue sections per mouse. All animal experiments were performed in compliance with the institutional guidelines (Ewha Womans University, Korea) for the care and use of laboratory animals.

### *In vitro* osteoclastogenesis

The non-adherent bone marrow-derived monocytes/macrophages (BMM) lineage cells derived from C57BL/6 mice were seeded and cultured in α-MEM (Invitrogen) containing 10% FBS and M-CSF (10 ng/ml, R&D systems). After 2 days, the nonadherent cells including lymphocytes were removed and the adherent cells were used as BMMs. The differentiation of BMM to osteoclast cells was induced by treating them with either soluble RANKL (50 ng/ml, Peprotech) or TNF-α (20 ng/ml) in the presence of M-CSF. After 5 days of induction, the cells were fixed and stained for tartrate-resistant acid phosphatase (TRAP). The cells were observed using a Zeiss Axiovert 200 microscope (Carl Zeiss) equipped with a plan-Neofluor objective lens. The images were analyzed using AxioVision 3.1 software (Carl Zeiss). The TRAP-positive multinucleated (>3 nuclei) cells were counted as osteoclast-like cells.

### Statistics

Data were analyzed using Student's *t*-test on SigmaPlot 8.0 software. The *P* values were derived to assess statistical significance and indicated on figure panels.

## Supporting Information

Figure S1 Action of Hsp60-specific antisense oligodeoxynucleotide (AS-ODN). A. Schematic representation of two different Hsp60 AS-ODNs. B. Hsp60 expression in mock- or ODN-transfected HeLa cells. C. TNF-α-induced MAP kinase activation in HeLa cells transfected with either mock or ODNs. The activation was analyzed by using phospho-specific antibodies. The phosphoblots were re-probed with whole protein antibodies for equal loading. D. Activation of various transcription factors in mock- or ODN-transfected HeLa cells. AP-1 and NF-AT transcriptional activation were induced by epidermal growth factor (EGF, 100 ng/ml). CRE transcriptional activation was induced by forskolin (1 µM). E. TNF-α-induced NF-κB transcriptional activation in 293T and A549 cells transfected with either mock or ODNs as indicated. In D and E, the relative luciferase activity was measured using an enhanced luciferase assay kit (Promega) and normalized to the β-galactosidase activity. Data are means ± S.D. of four independent experiments (In E, *P<0.001 and **P<0.05 versus stimulated S-ODN-transfected cells).
(2.28 MB EPS)

Figure S2 Selective role of cytosolic Hsp60 (Hsp60c) in IKK/NF-κB signaling. A. TNF-α-induced JNK activation in HeLa cells expressing Hsp60c (HA tag). B - D. Activation of various transcription factors in HeLa cells transfected with either control vector or Hsp60c (HA tag). AP-1 (B) and NF-AT (C) transcriptional activation were induced by epidermal growth factor (EGF, 100 ng/ml). CRE transcriptional activation (D) was induced by forskolin (1 µM). The relative luciferase activity was measured using an enhanced luciferase assay kit (Promega) and normalized to the β-galactosidase activity. Data are means ± S.D. of three independent experiments.
(1.35 MB EPS)

Figure S3 DEN induces hepatic cell apoptosis. The four-week-old C57BL/6j male mice were intraperitoneally injected with DEN (10 mg/kg). After the indicated time periods of DEN treatment, animals were sacrificed and processed to prepare tissue sections and images as described in Experimental Procedures. TUNEL positive cells were counted in three tissue sections per mouse. Representative images (A) are shown. Data in the quantitative graph (B) are mean ± S.D. of TUNEL positive cells per unit area.
(4.30 MB EPS)

Figure S4 Mitochondrial release of Hsp60 in staurosporine-treated HeLa cells. HeLa cells were treated with 1 µM staurosporine and then subjected to subcellular fractionation using the ProteoExtract subcellular proteome extraction kit (Roche). Peroxiredoxin-III (Prx III) and α-tubulin were used as mitochondrial and cytosolic markers, respectively. In particular, an antioxidant enzyme called Prx III (25-kDa in molecular size), which is present in the mitochondrial matrix, was useful for monitoring mitochondrial rupture. In western blots, the mitochondrial fractions were loaded at the volume of one-fifth of cytosolic fraction for appropriate comparison.
(1.31 MB EPS)

Figure S5 Cytosolic Hsp60 plays a significant survival role in RANK-mediated osteoclastogenesis. The ODN-pretreated BMM cells were treated with either RANKL (A) or TNF-α (B) for 5 days in the presence of M-CSF. The TRAP-positive multinucleated osteoclast cells were counted as described in the Experimental Procedures. Data represent the means±SD of triplicate from one of two independent sets of experiments, all of which showed similar results (* P<0.02 versus the stimulated sense-ODN). Representative pictures are shown.
(7.72 MB EPS)
